# Circadian Phase Modulates Embryonic Susceptibility to Bisphenol A-Induced ASD-Related Behavioral Alterations via *nr1d1*

**DOI:** 10.3390/toxics14060485

**Published:** 2026-05-31

**Authors:** Ying Wu, Jianjun Liu, Pinyi Chen, Xinwei Wang, Yuting Wang, Xiaoyao Song, Jie Zhang

**Affiliations:** 1 Soochow University Medical College, Soochow University, Suzhou 215123, China; wuying@suda.edu.cn; 2 Liyang City Center for Disease Control and Prevention, Changzhou 213000, China; 20205247050@alu.suda.edu.cn; 3 Department of Toxicology, School of Public Health, Medical College, Soochow University, Suzhou 215123, China; chenpy0219@163.com (P.C.); w19853146632@163.com (X.W.); 13206462812@163.com (Y.W.)

**Keywords:** bisphenol A, circadian phase, *nr1d1*, embryonic exposure, ASD-related behaviors

## Abstract

Emerging evidence links environmental exposures and circadian dysregulation to autism spectrum disorder (ASD), yet whether circadian phase modulates vulnerability to developmental toxicants remains unclear. Here, we investigated whether embryonic bisphenol A (BPA) exposure induces circadian phase-dependent ASD-related behavioral alterations via disruption of *nr1d1* rhythmicity in zebrafish. In control larvae, *nr1d1* exhibited significant circadian oscillation, whereas BPA exposure reduced expression levels and dampened oscillation amplitude. Two-way ANOVA revealed significant treatment × phase interactions in *nr1d1* expression. Pharmacological activation of Nr1d1 partially restored rhythmic expression. Behavioral assessments conducted at defined circadian phases demonstrated a significant treatment × phase interaction in social preference. BPA-exposed larvae exhibited reduced social preference selectively at circadian time 15 (CT15), corresponding to the trough phase of *nr1d1* expression, whereas no differences were observed at circadian time 3 (CT3). In contrast, tactile hyper-responsiveness showed a significant treatment effect but no phase interaction. BPA exposure also induced phase-dependent alterations in ASD-related genes, including *α-nrxn2a* and *β-nrxn3a,* with significant treatment × phase interactions. At the molecular level, BPA increased reactive oxygen species, impaired antioxidant defense, enhanced neuroinflammatory responses, and disrupted excitatory–inhibitory balance. Several of these endpoints exhibited phase-dependent modulation and were partially attenuated by Nr1d1 activation. These findings indicate that circadian phase modulates embryonic susceptibility to BPA-induced ASD-related behavioral and molecular alterations. Disruption of nr1d1 rhythmicity may contribute to time-of-day-specific neurodevelopmental vulnerability following environmental exposure.

## 1. Introduction

Autism spectrum disorders (ASD) is a heterogeneous neurodevelopmental condition characterized by persistent deficits in social communication and restricted or repetitive behaviors [1]. The reported prevalence of ASD has increased over recent decades, reaching approximately 2% in some preschool cohorts [2]. Although genetic factors contribute substantially to ASD liability, the pace of prevalence change and accumulating evidence for gene–environment interactions suggest that environmental exposures may modulate risk in susceptible individuals [3,4]. Among endocrine-disrupting chemicals (EDCs), bisphenol A (BPA) is widely used in polycarbonate plastics and epoxy resins and is ubiquitously detected in human biological samples [5]. BPA can bind multiple hormone receptors, including estrogen, androgen, and thyroid hormone receptors, and has been associated with reproductive, metabolic, and neurodevelopmental alterations [6]. Epidemiological and biomonitoring studies have reported associations between prenatal BPA exposure and ASD-related behavioral traits, although causality remains unresolved [2,7,8,9,10]. The biological mechanisms through which early-life BPA exposure may contribute to ASD-related behavioral phenotypes remain incompletely understood.

Sleep disturbance and circadian dysregulation are highly prevalent in individuals with ASD, affecting an estimated 50-80% of cases [11]. Altered melatonin secretion, abnormal sleep–wake cycles, and time-of-day variation in symptom severity have been documented [12]. Beyond behavioral manifestations, molecular alterations in core clock genes have been reported across multiple levels of investigation. Transcriptomic profiling studies have identified differential expressions of circadian genes, including *BMAL1* and *CLOCK*, in individuals with ASD, with evidence suggesting more pronounced dysregulation in severe phenotypes [13]. In experimental systems, disruption of circadian rhythms during neurodevelopment alters the rhythmic expression of clock genes in the suprachiasmatic nucleus and cortex and is accompanied by persistent ASD-like molecular and behavioral alterations in adulthood [14]. Moreover, genetic manipulation of core clock components provides further mechanistic support: mutation of Bmal1 has been shown to induce autistic-like behaviors and cerebellar dysfunction in mice, effects that are partially reversible by mTORC1 inhibition [15]. These observations indicated that circadian dysregulation in ASD extends beyond secondary sleep disturbances and may involve fundamental alterations in molecular timekeeping mechanisms. Given that the circadian system temporally organizes cellular physiology and coordinates neurodevelopmental processes across the 24 h cycle [16,17], disruption of circadian timing during sensitive developmental windows may influence neural circuit formation and behavioral outcomes [18]. These observations support the concept that circadian phase may gate neurodevelopmental risk, potentially shaping the magnitude or expression of environmentally induced phenotypes.

Circadian regulation exerts broad control over functional systems that are critical for neurodevelopment. Redox homeostasis, inflammatory signaling, synaptic remodeling, and excitatory–inhibitory (E/I) balance are all subject to circadian modulation [19,20,21,22]. Oscillatory control of antioxidant capacity influences cellular resilience to oxidative stress, while rhythmic regulation of immune mediator shapes neuroinflammatory tone. In parallel, circadian timing contributes to the coordination of synaptic transmission and neurotransmitter homeostasis, processes directly linked to E/I balance and behavioral regulation. Disruption of circadian coordination may therefore impair the temporal alignment of these systems, potentially increasing vulnerability to environmental stressors during specific circadian phases.

Experimental studies indicated that BPA can interfere with components of the circadian system. BPA exposure alters the expression of core clock genes including *Bmal1*, *Per2*, and *Nr1d1* in hypothalamic neurons [23]. Gestational low-dose BPA exposure disrupts suprachiasmatic nucleus (SCN) neurogenesis and circadian activity patterns with transgenerational consequences in mice [24]. Among core circadian regulators, *nr1d1* (REV-ERBα) functions as an important transcriptional repressor involved in maintaining circadian rhythmicity, redox homeostasis, inflammatory regulation, and neuronal activity. In zebrafish larvae, BPA has been reported to destabilize ni1d1 via m^6^A-dependent mechanisms, resulting in disrupted locomotor circadian rhythms [25]. These findings suggest that BPA can perturb circadian regulation at both molecular and behavioral levels. However, whether embryonic BPA exposure creates circadian phase-specific vulnerability windows for ASD-like behavioral phenotypes remains unknown.

Here, we hypothesized that embryonic BPA exposure dampens *nr1d1* rhythmicity, weakening circadian coordination of neurodevelopmental processes and producing time-of-day-dependent behavioral deficits. Using zebrafish as a diurnal vertebrate model with well-characterized circadian entrainment [26,27], we implemented a phase-stratified design to distinguish light-driven effects from endogenous circadian regulation [28,29]. Behavioral and molecular endpoints were assessed at circadian phases corresponding to peak and trough nr1d1 expression. In addition, pharmacological activation of Nr1d1 was employed to probe the functional contribution of disrupted *nr1d1* signaling. This chronobiologically informed framework was designed to determine whether circadian disruption functions as a temporal gate for BPA-induced neurodevelopmental vulnerability.

## 2. Materials and Methods

### 2.1. Chemicals and Reagents

Bisphenol A (BPA, purity ≥ 99%) and GSK4112 (a pharmacological agonist of REV-ERB receptors) were purchased from Sigma-Aldrich (St. Louis, MO, USA). A 250 mM BPA stock solution was prepared in dimethyl sulfoxide (DMSO) and stored at −20 °C. A stock solution of GSK4112 was prepared in DMSO and diluted to a final concentration of 1 μmol/L. Working solutions were freshly prepared by diluting the stock solutions in E3 embryo medium containing 5 mM NaCl, 0.17 mM KCl, 0.33 mM CaCl_2_, and 0.33 mM MgSO_4_ according to standard zebrafish husbandry protocols [30]. The final DMSO concentration in all groups, including the solvent control, was adjusted to 0.1%. TRIzol reagent (Beyotime, Nanjing, China) was used for RNA extraction. cDNA was synthesized using the RevertAid First Strand cDNA Synthesis Kit (Thermo Fisher Scientific, Waltham, MA, USA). All other reagents were of analytical grade.

### 2.2. Zebrafish Husbandry and Embryo Collection

**Figure 1:**
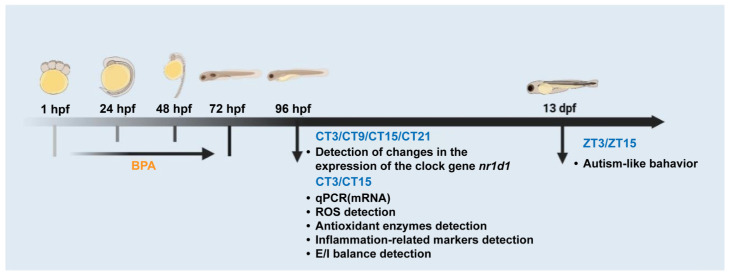
A workflow of the present study design.

The workflow of the present research is in Figure 1. Adult zebrafish (6–8 months old) were maintained in a recirculating aquaculture system at 28 ± 0.5 °C under a 14 h light:10 h dark photoperiod. Water parameters were monitored regularly and maintained within the following ranges: pH 7.0 ± 0.2, conductivity 500 ± 100 μS/cm, dissolved oxygen ≥ 6 mg/L, ammonia ≤ 0.02 mg/L, nitrite ≤ 0.02 mg/L, and nitrate approximately 20 mg/L. Fish were fed twice daily with brine shrimp.

For breeding, males and females were paired in spawning tanks in the evening and allowed to mate naturally the following morning. Fertilized eggs were collected, rinsed with system water, and examined under a stereomicroscope. Healthy embryos were selected and maintained in E3 embryo medium supplemented with methylene blue until experimental treatment.

### 2.3. BPA Exposure Paradigm and Circadian Sampling Design

Zebrafish embryos were randomly assigned to four experimental groups: solvent control (E3 medium containing 0.1% DMSO), GSK4112 (1 μmol/L), BPA (25 μmol/L), and BPA + GSK4112 (25 μmol/L + 1 μmol/L). This concentration was selected based on our previously published study [25] and in accordance with the OECD Fish Embryo Toxicity (FET) Test guideline (OECD TG236) [31]. Under our experimental conditions, this concentration corresponded to approximately half of the experimentally determined LC_50_ value and did not induce significant mortality or gross morphological abnormalities during embryonic development. Embryos were exposed from fertilization to 72 hpf, and treatment solutions were renewed every 24 h. At 72 hpf, larvae were transferred to fresh E3 medium and placed under constant darkness (DD). To assess endogenous circadian rhythms, larvae were maintained under DD from 72 hpf until sampling at 96 hpf to eliminate acute light-induced phase resetting.

Circadian time (CT) was defined relative to projected lights-on under DD conditions, with CT0 corresponding to subjective day onset. Based on preliminary rhythmic profiling of nr1d1 expression, CT3 and CT15 were selected to represent the subjective day and subjective night, respectively. All sampling under DD was conducted under dim red light.

Behavioral experiments were conducted at 13 dpf under a 14:10 light:dark (LD) photoperiod and expressed as Zeitgeber time (ZT), where ZT0 and ZT14 correspond to lights-on and lights-off, respectively. In contrast, molecular and biochemical assays were performed at 96 hpf under DD conditions and expressed as circadian time (CT) to assess endogenous rhythmic regulation independent of acute light-driven effects, consistent with established zebrafish circadian research paradigms [29].

For molecular analyses, larvae were sampled at CT3 and CT15 at 96 hpf. At each time point, larvae were rapidly euthanized under dim red light, rinsed briefly, and dissected to separate heads from trunks when required. Tissues were snap-frozen in liquid nitrogen and stored at −80 °C until analysis. The number of larvae used for each assay is specified in the corresponding sections below.

### 2.4. Assessment of ASD-Related Behaviors in Zebrafish Larvae

Social preference-related behavior was evaluated using a custom-designed behavioral apparatus as previously described (Supplementary Figure S3A). The apparatus was placed within a DanioVision behavioral tracking system (Noldus, Wageningen, The Netherlands). Each experimental group consisted of 10 independent larvae (biological replicates). Larvae were individually placed in the free-swimming test chamber, and locomotor activity was recorded for 60 min under constant illumination. Behavioral parameters related to social interaction were quantified using the accompanying tracking software according to predefined zone settings. For tactile response assessment, a separate custom-designed setup was used (Supplementary Figure S3B). Following tactile stimulation, swimming behavior was recorded for 60 min under constant illumination. Behavioral responses were analyzed to assess sensory reactivity.

### 2.5. Quantitative Real-Time PCR Analysis

Total RNA was extracted from pooled larval heads using TRIzol reagent according to the manufacturer’s instructions. For each biological replicate, 60 larval heads were pooled as one sample. Three independent biological replicates were analyzed per group at each time point. RNA concentration and purity were determined using a NanoDrop spectrophotometer (Thermo Fisher Scientific, USA), and samples with A260/A280 ratios between 1.8 and 2.1 were used for further analysis. For cDNA synthesis, 1000 ng of total RNA was reverse-transcribed using the RevertAid First Strand cDNA Synthesis Kit (Thermo Fisher Scientific, USA) following the manufacturer’s protocol. Quantitative real-time PCR (qPCR) was performed using a QuantStudio Real-Time PCR System (Thermo Fisher Scientific, USA) with SYBR Green qPCR Master Mix. The thermal cycling conditions were as follows: initial denaturation at 95 °C for 2 min, followed by 45 cycles of 95 °C for 20 s and 60 °C for 40 s. Melt curve analysis was performed to confirm amplification specificity. β-actin was used as the internal reference gene. Each biological sample was analyzed in technical triplicate. Primer sequences are listed in Supplementary Table S2. Relative gene expression levels were calculated using the 2^(−ΔΔCT)^ method.

### 2.6. Measurement of Reactive Oxygen Species (ROS) Levels

At 96 hpf, larvae were sampled at two designated circadian time points under DD conditions. For each treatment group at each time point, six larvae were randomly selected and individually transferred into 24-well plates (one larval per well) containing 1 mL of system water. Larvae were incubated with DCFH-DA fluorescent probe at a final concentration of 20 μM and maintained at 28.5 °C for 30–40 min in the dark. After incubation, larvae were rinsed thoroughly with system water to remove excess probe. Fluorescence images were captured using a fluorescence microscope under identical exposure parameters for all groups. Prior to the imaging and fluorescence quantification procedures, a randomization process was implemented, whereby all samples were assigned a unique code by an independent investigator. The experimenters responsible for image acquisition and ImageJ-based analysis were also blinded to treatment allocation until the conclusion of the statistical analysis. Subtraction of background fluorescence was performed prior to analysis, and fluorescence intensity was subsequently normalized to the area of the head region of each larva. This was done to minimize variability caused by differences in larval size and imaging background. The head-region fluorescence intensity of individual larvae was subjected to quantitative analysis using ImageJ software (version 1.54f, NIH, Bethesda, MD, USA).

### 2.7. Analysis of Antioxidant Enzyme Activities

At 96 hpf, larvae were sampled at two designated circadian time points under DD conditions. For each treatment group at each time point, 40 larval heads were pooled as one biological replicate, and three independent biological replicates were analyzed. Larval heads were homogenized in ice-cold phosphate-buffered saline and centrifuged to obtain supernatants for biochemical assays. The activities of glutathione peroxidase (GSH-Px), glutathione S-transferase (GST), and superoxide dismutase (SOD) were determined using commercial enzyme activity assay kits according to the manufacturer’s instructions. Enzyme activities were normalized to total protein content.

### 2.8. Assessment of Astrocyte Activation and Neutrophil Recruitment

Transgenic zebrafish embryos Tg (*gfap*: EGFP) and Tg (*mpo*: EGFP) were screened under a stereomicroscope and randomly distributed into six-well plates (30 embryos per well, three wells per treatment group). Embryos were maintained at 28.5 ± 0.5 °C under a 14 h light:10 h dark photoperiod. Embryos were continuously exposed to the designated treatments until 72 hpf, after which they were transferred to fresh system water. From 72 hpf onward, larvae were maintained under DD conditions until sampling at 96 hpf. Fluorescence imaging was performed at CT3 and CT15. For each treatment group at each circadian time point, six larvae were randomly selected and imaged. All fluorescence images were acquired using identical microscope exposure settings across groups. Prior to imaging and fluorescence quantification, larvae were coded by an independent investigator, and the experimenters performing image acquisition and ImageJ analysis were blinded to treatment allocation. Background fluorescence was subtracted before analysis, and fluorescence intensity was normalized to the corresponding area to minimize variability associated with larval size and background signals. Head-region fluorescence intensity of neutrophils and astrocytes was quantified using ImageJ software (NIH, USA).

### 2.9. Observation of GABAergic Neurons

Transgenic zebrafish embryos Tg(*dlx5a/6a*:EGFP) were screened under a stereomicroscope and randomly distributed into six-well plates (30 embryos per well, three wells per treatment group). Embryos were maintained at 28.5 ± 0.5 °C under a 14 h light:10 h dark photoperiod and exposed to the designated treatments until 72 hpf. From 72 hpf onward, larvae were maintained under DD conditions until sampling at 96 hpf. Fluorescence imaging was performed at CT3 and CT15. For each treatment group at each circadian time point, six larvae were randomly selected for imaging. Images were acquired under identical exposure settings across all groups. Prior to fluorescence imaging and quantification, samples were coded, and investigators performing image acquisition and ImageJ analysis were blinded to treatment groups. Background fluorescence was subtracted before analysis, and fluorescence intensity was normalized to the corresponding ROI area to minimize technical variability associated with larval size and imaging conditions. Fluorescence intensity of GABAergic neurons was quantified using ImageJ software (NIH, USA).

### 2.10. Quantification of Glutamate and GABA Levels

At 96 hpf, larvae were sampled at CT1 and CT13 under DD conditions. For each treatment group at each time point, 90 larvae were pooled as one biological replicate, and four independent biological replicates were analyzed. Samples were snap-frozen in liquid nitrogen and stored at −80 °C until analysis. For metabolite extraction, samples were homogenized in 50 μL MP buffer and mixed with 200 μL methanol–acetonitrile (1:1, *v*/*v*). Following cryosonication, samples were centrifuged at 14,000 rpm for 20 min at 4 °C. Supernatants were lyophilized, reconstituted in the initial mobile phase, and subjected to UHPLC–MS analysis. Chromatographic separation was performed using an Agilent 1290 UHPLC system. The mobile phases consisted of 25 mM ammonium formate with 0.1% formic acid (A) and acetonitrile with 0.1% formic acid (B). The flow rate was 0.30 mL/min, injection volume 2 μL, and column temperature 45 °C. The gradient program was as follows: 0–18 min, 90–40% B (linear); 18.0–18.1 min, 40–90% B (linear); 18.1–23.0 min, 90% B (isocratic). Mass spectrometric detection was performed on a 5500 QTRAP system operating in positive electrospray ionization mode. Glutamate and GABA were quantified using targeted multiple reaction monitoring (MRM).

### 2.11. Statistical Analysis

All statistical analyses were performed using GraphPad Prism 9.5 (GraphPad Software, USA). Fluorescence intensity was quantified using ImageJ (NIH, USA). Data are presented as mean ± SEM. For *nr1d1* rhythmic profiling experiments involving four circadian time points (CT3, CT9, CT15, and CT21), circadian rhythmicity was evaluated using JTK_CYCLE (*p* < 0.05). Differences among treatments and time points were analyzed using two-way ANOVA followed by Tukey’s multiple comparisons test. For molecular, biochemical, and behavioral experiments conducted at two circadian phases (CT3 and CT15), data were analyzed using two-way ANOVA with treatment and circadian phase as independent factors, followed by Tukey’s post hoc test. A two-tailed *p*-value < 0.05 was considered statistically significant.

## 3. Results

### 3.1. Embryonic BPA Exposure Disrupts Circadian nr1d1 Expression

**Figure 2:**
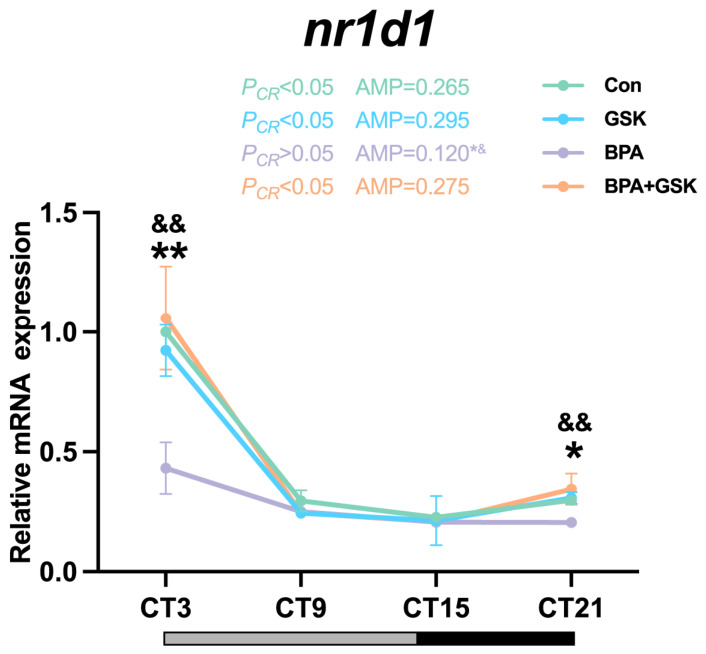
Effects of embryonic BPA exposure on circadian *nr1d1* expression in zebrafish larvae. Relative *nr1d1* mRNA levels were measured at CT3, CT9, CT15, and CT21. Data are shown as mean ± SEM (*n* = 3 independent biological replicates, each consisting of 60 pooled larvae). Expression levels were analyzed by two-way ANOVA followed by Tukey’s multiple comparisons test. Amplitude was analyzed by one-way ANOVA. Circadian rhythmicity was determined using JTK_CYCLE. P_CR_ denotes the JTK_CYCLE-derived *p*-value for circadian rhythmicity in each treatment group, and AMP represents the estimated oscillation amplitude. * *p* < 0.05, ** *p* < 0.01 vs. Con; ^&^ *p* < 0.05, ^&&^ *p* < 0.01 vs. BPA+GSK.

As shown in Figure 2, *nr1d1* mRNA exhibited significant circadian rhythmicity in control larvae (JTK_CYCLE, *p* < 0.05). BPA exposure reduced expression levels, particularly at CT3 and CT21. Two-way ANOVA revealed significant main effects of treatment and circadian phase, as well as a significant treatment × phase interaction (all *p* < 0.05). Post hoc analysis demonstrated significant differences between BPA and control groups at CT3 and CT21. Oscillation amplitude was significantly decreased in the BPA group compared with controls (one-way ANOVA, *p* < 0.05). Co-treatment with GSK4112 significantly attenuated the BPA-induced reduction in expression and oscillation amplitude.

### 3.2. Embryonic BPA Exposure Induces Circadian Phase-Dependent Social Preference Deficits and Alters Autism-Related Gene Expression

**Figure 3:**
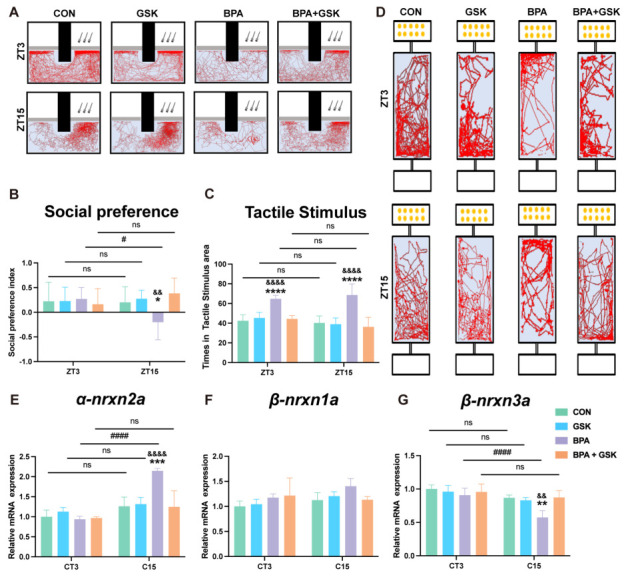
Effects of embryonic BPA exposure on social behavior, tactile responsiveness, and autism-related gene expression in zebrafish larvae. Behavioral assessments were performed at CT3 and CT15, corresponding to the trough and peak phases of *nr1d1* rhythmic expression. (**A**) Representative tracking images from the social preference assay. (**B**) Quantification of social preference index. (**C**) Representative tracking images from the tactile stimulus assay. (**D**) Quantification of the percentage of time spent in the tactile stimulus area. (**E**–**G**) Relative mRNA expression levels of *α-nrxn2a*, *β-nrxn1a*, and *β-nrxn3a* at CT3 and CT15. Data are presented as mean ± SEM (behavioral assays: *n* = 10 larvae per group; gene expression: *n* = 3 independent biological replicates, each consisting of 60 pooled larvae). Data were analyzed by two-way ANOVA followed by Tukey’s multiple comparisons test. * *p* < 0.05, ** *p* < 0.01, *** *p* < 0.001 vs. Con; ^&^ *p* < 0.05, ^&&^ *p* < 0.01, ^&&&^ *p* < 0.001, ^&&&&^ *p* < 0.0001 vs. BPA+GSK; ^#^ *p* < 0.05, ^##^ *p* < 0.01, ^###^ *p* < 0.001, ^####^ *p* < 0.0001 indicate comparisons between CT3 and CT15 within the same treatment group. ns indicates no statistical difference comparisons between CT3 and CT15 within the same treatment group.

As shown in Figure 3, behavioral assessments were conducted at CT3 and CT15, corresponding to the peak and trough phases identified from *nr1d1* rhythmic expression. Two-way ANOVA was used to examine the effects of treatment and circadian phase. In the social interaction test (Figure 3A,B), a significant treatment × phase interaction was detected (*p* < 0.05). No significant differences among groups were observed at CT3. However, at CT15, BPA-exposed larvae exhibited a significant reduction in social preference compared with controls (*p* < 0.05), and this deficit was attenuated by GSK4112 co-treatment (*p* < 0.01 vs. BPA). Moreover, a significant difference between CT3 and CT15 was observed within the BPA group, whereas no circadian variation was detected in control larvae, indicating a phase-dependent social impairment induced by BPA. In the tactile stimulus assay (Figure 3C,D), a significant main effect of treatment was observed, while neither a significant phase effect nor a treatment × phase interaction was detected. BPA exposure significantly increased the percentage of time spent in the tactile stimulus area at both CT3 and CT15 compared with controls (*p* < 0.001), and this increase was reversed by GSK4112. No significant differences between CT3 and CT15 were observed within any group. Analysis of autism-related genes revealed selective molecular alterations (Figure 3E–G). For *α-nrxn2a*, a significant treatment × phase interaction was identified. BPA exposure markedly increased expression at CT15 compared with controls (*p* < 0.001), and this elevation was attenuated by GSK4112. A significant CT1–CT13 difference was detected within the BPA group, whereas no circadian variation was observed in controls. Similarly, *β-nrxn3a* expression showed a significant interaction, with BPA significantly reducing expression at CT15 (*p* < 0.01 vs. Con), which was restored by co-treatment; a significant CT3-CT15 difference was present only in the BPA group. In contrast, *β-nrxn1a* expression showed no significant main effects or interaction at either time point.

### 3.3. Embryonic BPA Exposure Induces Oxidative Stress and Impairs Antioxidant Defense

**Figure 4:**
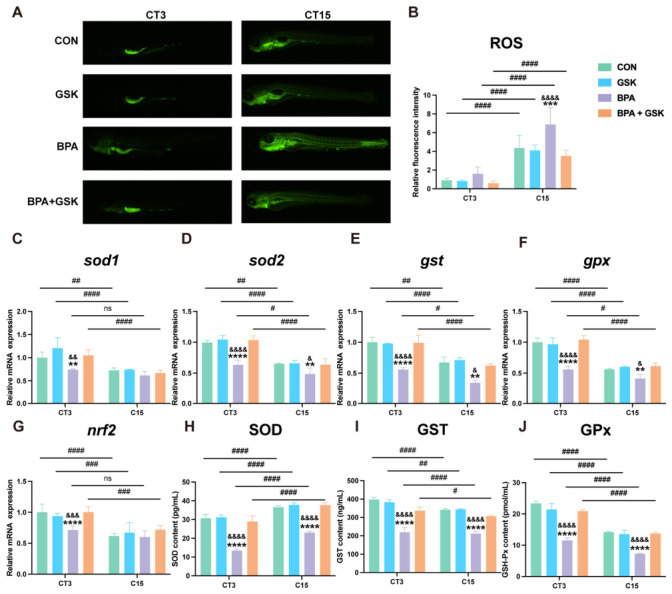
Effects of embryonic BPA exposure on oxidative stress and antioxidant defense in zebrafish larvae. Measurements were performed at CT3 and CT15, corresponding to two circadian phases. (**A**) Representative fluorescence images of ROS levels detected by DCFH-DA staining. (**B**) Quantification of ROS fluorescence intensity. (**C**–**G**) Relative mRNA expression levels of *sod1, sod2, gst, gpx*, and *nrf2* at CT3 and CT15. (**H**–**J**) Activities of antioxidant enzymes SOD, GST, and GPx at CT3 and CT15. Data are presented as mean ± SEM (gene expression: *n* = 3 independent biological replicates, each consisting of 60 pooled larvae; enzyme activity assays: *n* = 3 independent biological replicates, each consisting of 40 pooled larvae). Data were analyzed by two-way ANOVA followed by Tukey’s multiple comparisons test. * *p* < 0.05, ** *p* < 0.01, *** *p* < 0.001 vs. Con; ^&^ *p* < 0.05, ^&&^ *p* < 0.01, ^&&&^ *p* < 0.001, ^&&&&^ *p* < 0.0001 vs. BPA+GSK; ^#^ *p* < 0.05, ^##^ *p* < 0.01, ^###^ *p* < 0.001, ^####^ *p* < 0.0001 indicate comparisons between CT3 and CT15 within the same treatment group. ns indicates no statistical difference comparisons between CT3 and CT15 within the same treatment group.

As shown in Figure 4A,B, two-way ANOVA revealed significant main effects of treatment and circadian phase on ROS levels, as well as a significant treatment × phase interaction (*p* < 0.05). ROS fluorescence intensity was significantly higher at CT15 than at CT3 in all groups, indicating a significant difference between CT3 and CT15. BPA exposure markedly increased ROS levels at both CT3 and CT15 compared with controls (*p* < 0.001), and this increase was significantly attenuated by GSK4112. As shown in Figure 4C–G, antioxidant-related genes were significantly affected by treatment and circadian phase. BPA exposure significantly decreased the expression of *sod1*, *sod2*, *gst*, *gpx*, and *nrf2* at both CT3 and CT15 compared with controls. A significant difference between CT3 and CT15 was observed in most groups. However, for *sod1* and *nrf2*, this difference between CT3 and CT15 was not detected in the BPA group, whereas it remained significant in the other groups. As shown in Figure 4H–J, SOD, GST, and GPx activities exhibited significant main effects of treatment and circadian phase. Enzyme activities were significantly reduced in the BPA group at both CT3 and CT15 (*p* < 0.001). A significant difference between CT3 and CT15 was observed in all groups. GSK4112 partially restored antioxidant enzyme activities.

### 3.4. Embryonic BPA Exposure Enhances Neuroinflammatory Responses

**Figure 5:**
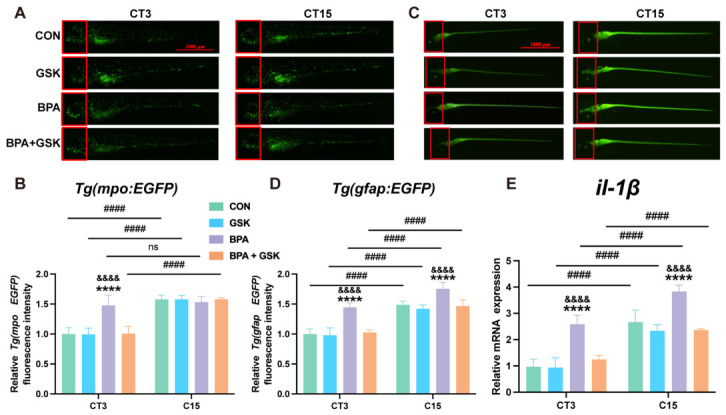
Effects of embryonic BPA exposure on neuroinflammatory responses in zebrafish larvae. Measurements were performed at CT3 and CT15, corresponding to two circadian phases. (**A**) Representative fluorescence images of neutrophils in Tg (*mpo*:EGFP) larvae. (**B**) Quantification of neutrophil fluorescence intensity. (**C**) Representative fluorescence images of astrocytes in Tg (*gfap*:EGFP) larvae. (**D**) Quantification of astrocyte fluorescence intensity. (**E**) Relative mRNA expression level of *il-1β* at CT3 and CT15. Data are presented as mean ± SEM (fluorescence imaging: *n* = 6 larvae per group; gene expression: *n* = 3 independent biological replicates, each consisting of pooled larvae). Data were analyzed by two-way ANOVA followed by Tukey’s multiple comparisons test. * *p* < 0.05, ** *p* < 0.01, *** *p* < 0.001 vs. Con; ^&^ *p* < 0.05, ^&&^ *p* < 0.01, ^&&^ *p* < 0.001 vs. BPA; ^#^ *p* < 0.05, ^##^ *p* < 0.01, ^###^ *p* < 0.001 indicate comparisons between CT3 and CT15 within the same treatment group. The red boxed region indicates the head-region ROI used for fluorescence quantification.

As shown in Figure 5A–D, neutrophil (Tg(*mpo*:EGFP)) and astrocyte (Tg(*gfap*:EGFP)) fluorescence intensities were significantly affected by treatment and circadian phase. BPA exposure significantly increased fluorescence intensity in both transgenic lines compared with controls (*p* < 0.001), and this elevation was significantly reduced by GSK4112. A significant difference between CT3 and CT15 was observed in the CON, GSK, and BPA+GSK groups, whereas this difference was not detected in the BPA group. Consistently, *il-1β* mRNA expression (Figure 5E) showed significant main effects of treatment and circadian phase. BPA exposure significantly upregulated *il-1β* expression at both CT3 and CT15 compared with controls (*p* < 0.001). GSK4112 significantly reduced the BPA-induced increase in *il-1β* expression. A significant difference between CT3 and CT15 was observed in all groups.

### 3.5. Embryonic BPA Exposure Alters E/I Balance-Related Parameters

**Figure 6:**
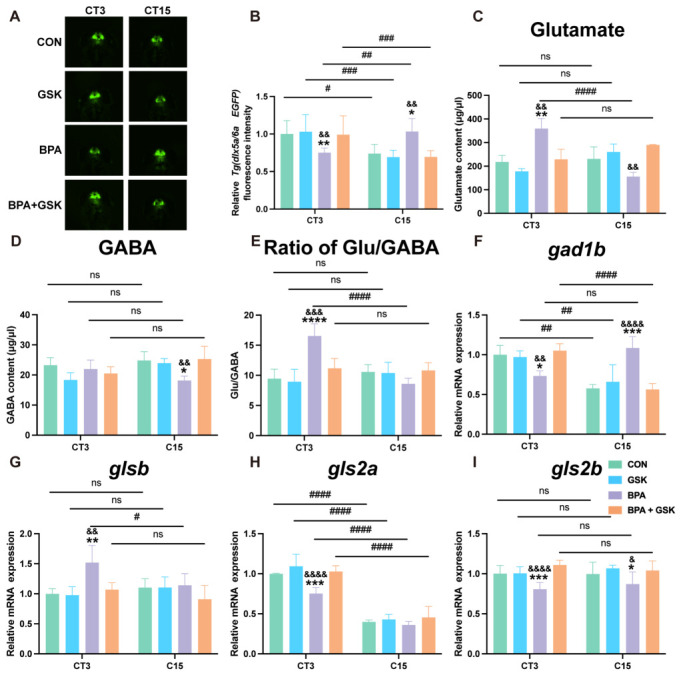
Embryonic BPA exposure alters E/I balance-related parameters in zebrafish larvae. Measurements were performed at CT3 and CT15, corresponding to two circadian phases. (**A**) Representative fluorescence images of GABAergic neurons in Tg (*dlx5a/6a*:EGFP) larvae. (**B**) Quantification of relative Tg (*dlx5a/6a*:EGFP) fluorescence intensity (*n* = 6 larvae per group). (**C**) Glutamate content. (**D**) GABA content. (**E**) Ratio of Glu/GABA. Neurotransmitter measurements were performed using pooled samples (90 larvae per biological replicate, *n* = 3 independent biological replicates). (**F**–**I**) Relative mRNA expression levels of E/I-related synthetic genes, including *gad1b* (**F**), *glsb* (**G**), *gls2a* (**H**), and *gls2b* (**I**) (*n* = 3 independent biological replicates). Data are presented as mean ± SEM. Statistical analyses were performed using two-way ANOVA followed by Tukey’s multiple comparisons test. * *p* < 0.05, ** *p* < 0.01, *** *p* < 0.001 vs. Con; ^&^ *p* < 0.05, ^&&^ *p* < 0.01, ^&&^ *p* < 0.001, ^&&&&^ *p* < 0.0001 vs. BPA; ^#^ *p* < 0.05, ^##^ *p* < 0.01, ^###^ *p* < 0.001, ^####^ *p* < 0.0001 indicate comparisons between CT3 and CT15 within the same treatment group. ns indicates no statistical difference comparisons between CT3 and CT15 within the same treat-ment group. The red hollow square indicates the head-region ROI used for fluorescence quantification.

As shown in Figure 6A,B, Tg(*dlx5a/6a*:EGFP) fluorescence intensity was significantly affected by treatment and circadian phase. BPA exposure significantly decreased fluorescence intensity at CT3 compared with the control group (*p* < 0.01). GSK4112 treatment significantly increased fluorescence intensity compared with the BPA group at CT3. A significant difference between CT3 and CT15 was observed in all groups. Neurotransmitter measurements further revealed treatment- and phase-dependent alterations (Figure 6C–E). At CT3, BPA exposure significantly increased glutamate levels compared with controls (*p* < 0.01), and this increase was significantly reduced by GSK4112. At CT15, glutamate levels in the BPA group were significantly lower than those at CT3. GABA levels did not differ among groups at CT3; however, at CT15, BPA exposure significantly reduced GABA levels compared with controls (*p* < 0.05), and GSK4112 significantly increased GABA levels compared with the BPA group. Consistently, the Glu/GABA ratio was significantly elevated in the BPA group at CT3 compared with controls (*p* < 0.001). A significant difference between CT3 and CT15 was observed in the BPA group for glutamate levels and the Glu/GABA ratio. Analysis of E/I-related synthetic genes revealed significant treatment- and phase-dependent alterations (Figure 6F–I). At CT3, BPA exposure significantly decreased the mRNA expression of *gad1b* (*p* < 0.05), *gls2a* (*p* < 0.001), and *gls2b* (*p* < 0.001) compared with the control group, whereas *glsb* expression was significantly increased (*p* < 0.01). GSK4112 treatment significantly increased gad1b expression at CT3 and *gls2b* expression at CT15 compared with the BPA group. With respect to circadian phase, significant differences between CT3 and CT15 were observed for *gad1b* and *gls2a* expression. A phase-dependent difference was also detected for *glsb* expression in the BPA group, whereas no significant phase-dependent difference was observed for *gls2b* expression.

## 4. Discussion

ASD has been increasingly linked to exposure to environmental EDCs such as BPA [7,32,33]. In the present study, we demonstrate that embryonic BPA exposure induces phase-dependent social preference deficits in zebrafish larvae, with impairments more pronounced at ZT15, corresponding to the trough of *nr1d1* expression, than at ZT3. This finding indicates that behavioral vulnerability is not static but varies across the circadian cycle. Such temporal modulation is consistent with clinical evidence suggesting that ASD-related behaviors fluctuate across the day–night cycle rather than remaining constant [11,34]. Mechanistically, BPA reduced the oscillation amplitude of *nr1d1* and disrupted the phase-dependent expression of autism-related genes, indicating that developmental exposure compromises circadian regulatory mechanisms rather than exerting uniform effects across the circadian cycle. The dampening of *nr1d1* rhythmicity observed here is consistent with previous evidence that BPA destabilizes *nr1d1* transcripts via m^6^A-dependent mechanisms, resulting in reduced locomotor rhythm amplitude [25]. These findings suggest that BPA-induced circadian disruption may not be limited to locomotor rhythm alterations but may also contribute to phase-dependent social preference vulnerability.

Redox regulation exhibited robust circadian organization in control larvae. Antioxidant defenses including GPx, GST, SOD, and the transcription factor *nrf2* peaked during the subjective day (CT3), when reactive oxygen species (ROS) were lowest, consistent with the anticipatory arrangement reported in mammals [35,36,37,38]. Such temporal coordination is consistent with a clock-controlled buffering strategy in which protective capacity rises in advance of anticipated oxidative demand. Embryonic BPA exposure did not uniformly elevate oxidative stress; rather, it attenuated rhythmic amplitude by reducing daytime antioxidant peaks and alters the temporal balance between ROS production and antioxidant defense. Consequently, oxidative burden became temporally concentrated at CT15, a phase intrinsically characterized by low *nr1d1* expression. The temporal alignment between heightened ROS levels and behavioral impairment suggests that BPA disrupts circadian redox gating, thereby amplifying vulnerability within an already permissive nighttime window. It is likely that oxidative stress and impaired antioxidant defense form a reciprocal feedback loop that collectively contributes to BPA-induced neurodevelopmental toxicity. This interpretation is consistent with evidence that BPA induces oxidative stress [39] and that redox imbalance is implicated in ASD pathology [40,41]. The restoration of rhythmic amplitude and oxidative balance by GSK4112 further supports the notion that maintaining *nr1d1* rhythmicity stabilizes the redox system. Although oxidative stress alterations were temporally associated with behavioral abnormalities and neuroinflammatory activation, direct causal relationships remain to be further validated.

Excessive neuroinflammatory activation during neurodevelopment has been reported to impair synaptic pruning, neuronal connectivity, and E/I balance, thereby contributing to ASD-related neurobehavioral abnormalities. Baseline neuroinflammatory activity was similarly under circadian control. In untreated larvae, neutrophil recruitment, *il-1β* expression, and astrocyte activation displayed coordinated oscillations, with higher levels during the subjective night and lower levers during the day, consistent with reports that inflammatory processes are temporally gated by the circadian clock [42,43]. Such restriction likely confines inflammatory signaling to discrete windows and prevents sustained neural exposure. BPA exposure elevated baseline inflammatory activity and exaggerated nighttime peaks, effectively flattening rhythmic regulation while increasing overall inflammatory burden. Maximal dysregulation occurred at CT15 and paralleled behavioral severity, in agreement with evidence linking both BPA and ASD to neuroinflammation [44,45,46,47]. Given that Nr1d1 proteins act as transcriptional repressors of inflammatory gene programs, attenuation of nr1d1 rhythmicity may weaken this temporal brake, allowing inflammatory activity to escape circadian confinement during development. GSK4112 restored both amplitude and phase-dependent inflammatory patterns, supporting a mechanism in which *nr1d1* constrains inflammatory tone in a time-of-day-specific manner.

Excitatory/inhibitory (E/I) balance also demonstrated circadian coordination. In control zebrafish, glutamate, GABA, and their synthesizing enzymes (*glsb*, *gls2a*, *gls2b*, and *gad1b*), as well as GABAergic neuronal activity, exhibited oscillatory patterns, yet the overall Glu/GABA ratio remained relatively stable across phases, reflecting clock-coordinated neurotransmitter regulation [19,48]. Embryonic BPA exposure disrupted this temporal organization in a phase-specific and bidirectional manner, increasing the E/I ratio during the subjective day and decreasing it during the subjective night. Notably, the decline in glutamate levels evident at CT15 was concomitant with the repression of gls2a/b expression subsequent to BPA exposure, given that GLS enzymes function as the catalysts for the transformation of glutamine to glutamate. These findings suggest that impaired glutamate synthesis may contribute to nighttime neurotransmitter dysregulation. However, the regulation of glutamate homeostasis is achieved through multiple interconnected processes, including neurotransmitter synthesis, synaptic release, neuronal uptake, metabolic recycling, and circadian modulation. Consequently, the altered neurotransmitter profiles evident in the present study are more likely to be indicative of a disruption in the wider neurochemical regulatory networks as opposed to alterations in a solitary metabolic enzyme in isolation. These time-locked imbalances paralleled behavioral abnormalities, suggesting that circadian misalignment of neurotransmitter programs contributes to the phenotype. As E/I imbalance is a central framework in ASD biology, with both hyperexcitable and hypoexcitable states reported across models [49,50], our findings introduce a temporal dimension by indicating that *nr1d1* rhythmicity helps synchronize molecular and circuit-level processes to maintain phase-appropriate neural balance. These findings suggest that neurotransmitter dysregulation may contribute to, but may not fully account for, the observed circadian phase-dependent behavioral abnormalities. Normalization of the Glu/GABA ratio by GS4112 further implicated *nr1d1*-dependent circadian control in coordinating synaptic homeostasis.

The zebrafish is an advantageous model for investigating developmental neurotoxicity and circadian regulation due to several factors. These include the rapid embryonic development, its optical transparency and the fact that the genes that control its circadian rhythm are highly conserved. Furthermore, the availability of transgenic fluorescent reporter lines enables real-time visualization of neuroinflammatory and neurotransmitter-related processes in vivo. Nevertheless, it is important to acknowledge the limitations of the zebrafish model. A comparison of the brain architecture of zebrafish with that of mammals reveals a simpler architecture in the former and reduced behavioral complexity. Consequently, complex social and cognitive behaviors that are relevant to ASD cannot be fully recapitulated in zebrafish. Consequently, although the present study provides mechanistic insights into circadian phase-dependent vulnerability to BPA exposure, further validation of the translational relevance of these findings is required, and this will be achieved by conducting future studies using mammalian models.

These findings support a model in which embryonic BPA exposure dampens *nr1d1* rhythmicity and disrupts temporal coordination across multiple physiological systems, leading to phase-specific behavioral alterations. The present study primarily focused on nr1d1 transcriptional rhythmicity, while broader circadian clock gene networks were not systematically investigated. Given the complex transcriptional feedback interactions among core circadian regulators, future studies incorporating additional clock genes and protein-level validation will be important to further elucidate the molecular mechanisms and functional consequences underlying BPA-induced circadian dysregulation. This model should be interpreted within the context of the experimental design. To differentiate endogenous circadian regulation from acute light-driven effects, behavioral assessments were conducted under LD conditions, whereas molecular analysis were performed under DD conditions, consistent with established zebrafish circadian research paradigms [29]. Moreover, the concentration of BPA employed exceeds typical environmental levels. It was selected to ensure reproducible neurodevelopmental effects under sublethal exposure conditions, without significant mortality or gross developmental malformations. The study focused on embryonic exposure and ASD-related behavioral phenotypes. Future studies employing environmentally relevant maternal exposure paradigms and incorporating sleep-related assessments will further clarify the developmental ASD-like behavioral outcomes associated with BPA exposure.

## 5. Conclusions

The present study demonstrates that embryonic BPA exposure attenuates *nr1d1* rhythmic amplitude and disrupts circadian coordination across redox, inflammatory, and neurotransmitter systems in zebrafish larvae. These alterations are associated with phase-dependent ASD-related behavioral changes, particularly during the subjective night. Together, these findings suggest that impaired circadian rhythmic regulation may represent a mechanistic pathway linking developmental BPA exposure to ASD-like phenotypes.

## Data Availability

The original contributions presented in this study are included in the article/Supplementary Materials. Further inquiries can be directed to the corresponding authors.

## Supplementary Materials

The following supporting information can be downloaded at: https://www.mdpi.com/article/10.3390/toxics14060485/s1.
