# Supplementary material for: Circadian Phase Modulates Embryonic Susceptibility to Bisphenol A-Induced ASD-Related Behavioral Alterations via nr1d1
Source: Toxics. 2026 May 31;14(6):485. doi: 10.3390/toxics14060485 (PMC13306773; doi:10.3390/toxics14060485)
Supplement: Supplementary file 1 [file toxics-14-00485-s001.zip › toxics-4300311-supplementary.pdf]

# Supplementary Material

## S1. BPA exposure of zebrafish embryos and sample collection

Fertilized zebrafish embryos were distributed into six-well plates at a density of 30–35 embryos per well in 3 mL E3 medium. Stock solutions were prepared in DMSO and added to achieve final concentrations of 25 μM BPA and/or 1 μM GSK4112, with solvent concentration kept constant across groups (0.01% DMSO, v/v). Each treatment was performed in triplicate wells.

Exposure was conducted from fertilization to 72 hpf, with daily renewal of treatment solutions. At 72 hpf, larvae were transferred to fresh E3 medium and maintained under constant darkness until 96 hpf. For circadian profiling of *nr1d1* expression, larvae were sampled at CT3, CT9, CT15, and CT21 under dim red light. Samples were immediately frozen in liquid nitrogen and stored at −80°C until analysis.

## S2. Primer information

**Table S2.** Primer sequences for target genes.

| Name | Primer type | Primer sequence（5–3’） |
|---|---|---|
| *β-actin* | Forward | ACGAACGACCAACCTAAACTCT |
| | Reverse | TTAGACAACTACCTCCCTTTGC |
| *nr1d1* | Forward | ACATCCCAGGGTTTAGCACG |
| | Reverse | GTTCGGAGAGCCCGGATTAG |
| *α-nrnx2a* | Forward | GCCTCCATGCACCTCTTCTT |
| | Reverse | TGTCATTGAGCGGCTTGTCG |
| *β-nrnx2a* | Forward | CAACGGATGGGACAAATGGG |
| | Reverse | GTGGTGCTCAGGCTGGAAGA |
| *nrf2* | Forward | AAGCAGACGGAGGAGGAG |
| | Reverse | GGAGGTGTTCAGGCAAGG |
| *gpx* | Forward | TCCTGCAGTCTCTGAAATACG |
| | Reverse | TGTACCTCTTGAATGGTTCCC |

| | | |
|---|---|---|
| *gst* | Forward | CCCTCGTCTGGACTCTTTC |
| | Reverse | CAGTTTCTTGAAGTTCTCGCA |
| *sod1* | Forward | CAAGAGGGTGAAAAGAAGCCA |
| | Reverse | GGTCACATTACCCAGGTCTCC |
| *sod2* | Forward | TGGACAAATCTGTCACCCAA |
| | Reverse | ACGCATGTTCCCCAGACATCTA |
| *il-1b* | Forward | GGCTGTGTGTTTGGGAATCT |
| | Reverse | TGATAAACCAACCGGGACA |
| *glsa* | Forward | AGATGCGGCCAATGACAAGA |
| | Reverse | CTTTCAGCGCCGTGGTAAAC |
| *glsb* | Forward | CTATAGGATGGAGACTGTTGGAGA |
| | Reverse | TGACCCGAATGAGAACCAG |
| *gls2a* | Forward | TTTCTGGGTTGGAGGACTTG |
| | Reverse | TGCAAACCTGTGCTTTTGAG |
| *gls2b* | Forward | CGACTACTCGGGACAGTTCG |
| | Reverse | CCAGTTCCTGACAGAAGCGA |
| *gad1b* | Forward | AACTCAGGCGATTGTTGCAT |
| | Reverse | TGAGGACATTTCCAGCCTTC |

**S3. The schematic diagram of the behavioral setup for zebrafish larvae**

The social behavior panel consisted of three areas: stimulation area, no-stimulation area, and free-swimming test area (Arena). The Arena was divided into two small areas: social zone and non-social zone. One fish from each group was selected and placed in the designated test area, with fish water added to the level of the board edge to allow the zebrafish larvae to swim freely (Figure S3A).

The tactile stimulation behavior panel consisted of three areas including brine-shrimp-stimulation area, no-brine-shrimp-stimulation area, and free-swimming test area. The free-swimming area was divided into two small areas: one near the brine-shrimp-stimulation area and the other near the no-brine-shrimp-stimulation area (Figure S3B). One fish from each group was selected and placed in the designated test area,

with fish water added to the level of the edge of the board to allow the zebrafish larvae to swim freely.

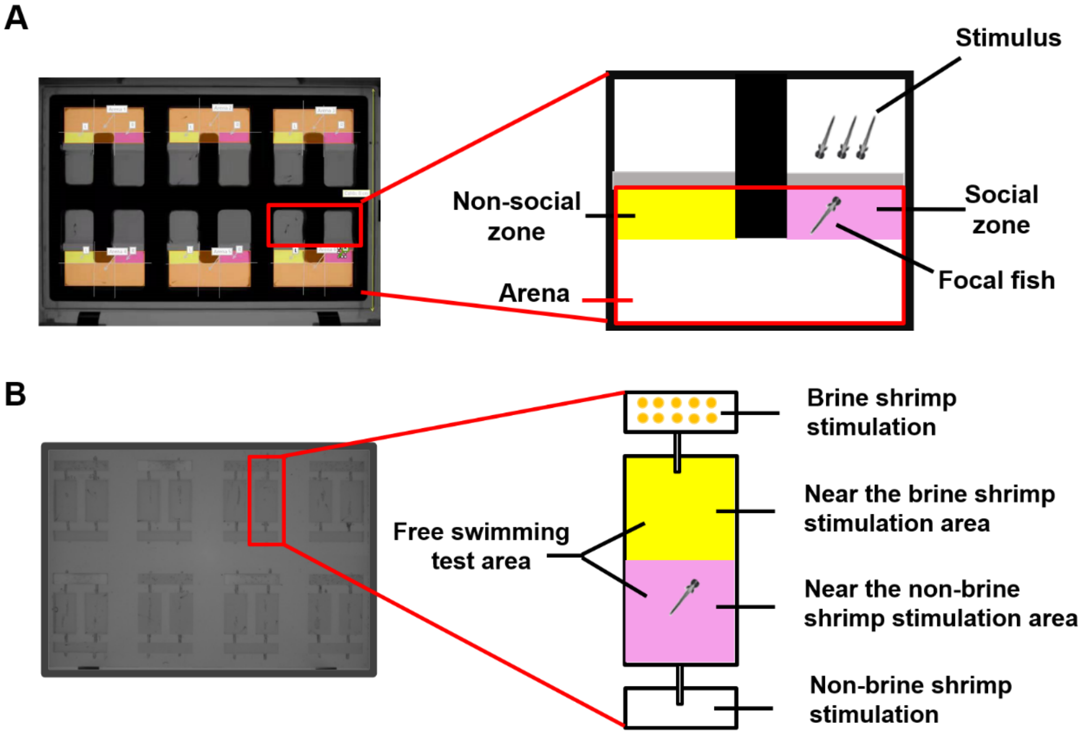


**Figure S3. The schematic diagram of the behavioral setup for zebrafish larvae.** **(A)** Zebrafish larvae social behavior board consisted of three areas: stimulation area, no-stimulation area, and free-swimming test area (Arena). The free-swimming area was divided into two small areas: social zone and non-social zone. One fish from each group was selected and placed in the designated test area, with fish water added to the level of the board edge to allow the zebrafish larvae to swim freely. **(B)** Zebrafish larvae tactile stimulation behavior board consisted of three areas including brine-shrimp-stimulation area, no-brine-shrimp-stimulation area, and free-swimming test area. The free-swimming area was divided into two small areas: one near the brine-shrimp-stimulation area and the other near the no-brine-shrimp-stimulation area. One fish from each group was selected and placed in the designated test area, with fish water added to the level of the edge of the board to allow the zebrafish larvae to swim freely.
